# Interleukin-37 does not confer protection against stone formation or kidney injury at the peak of disease in a murine calcium oxalate model

**DOI:** 10.1007/s00240-026-02061-7

**Published:** 2026-10-10

**Authors:** Nirmal P. Bhatt, Theresa T. H. Nguyen, Ina Rudloff, Bernadette Jones-Freeman, Guy J. M. Cameron, Gabriel R. Rodriguez, Marcel F. Nold, Aniruddh V. Deshpande, Claudia A. Nold-Petry, Malcolm Ronald Starkey

**Affiliations:** 1 Department of Immunology, School of Translational Medicine, Monash University, Melbourne, VIC Australia; 2 Bladder and Kidney Health Discovery Program, School of Translational Medicine, Monash University, Melbourne, VIC Australia; 3 The Ritchie Centre, Hudson Institute of Medical Research, Melbourne, VIC Australia; 4 Department of Paediatrics, Monash University, Melbourne, VIC Australia; 5 Priority Research Centre GrowUpWell, College of Health, Medicine and Wellbeing, Hunter Medical Research Institute, The University of Newcastle, Newcastle, NSW Australia; 6 Centre for Kidney Research, The Children’s Hospital at Westmead, The University of Sydney, Sydney, NSW Australia; 7 Urology Unit, Department of Surgery, The Children’s Hospital at Westmead, Sydney, NSW Australia; 8 School of Medical Sciences, Faculty of Health and Medicine, The University of Sydney, Sydney, NSW Australia; 9 Monash Newborn, Monash Children’s Hospital, Melbourne, Australia VIC

**Keywords:** Calcium oxalate, Kidney stone, Nephrolithiasis, Urolithiasis, Interleukin-37, IL-37

## Abstract

Calcium oxalate (CaOx) stones are the most prevalent form of nephrolithiasis in adults. While murine models of CaOx stone disease are established, many lack translational features for therapeutic evaluation, including in vivo assessment of kidney function and molecular profiling of renal inflammation. We refined the glyoxylate‑induced CaOx mouse model by incorporating non‑invasive transdermal glomerular filtration rate (*t*GFR) measurements to enable longitudinal assessment of kidney function. This platform was used to investigate the renoprotective role of the anti‑inflammatory cytokine interleukin‑37 (IL‑37). Because mice do not express IL‑37, we used IL‑37 transgenic (IL‑37tg) mice that express the human *IL37b* isoform. Male wild‑type and IL‑37tg mice received daily intraperitoneal glyoxylate to induce CaOx deposition. Crystal burden and tubular injury were quantified using digital image analysis of whole‑kidney sections. Kidney function was assessed using *t*GFR and validated by serum creatinine, blood urea nitrogen (BUN), and symmetric dimethylarginine (SDMA). Renal inflammation was profiled using an RT² Profiler PCR Array, with key transcripts validated by qPCR. CaOx deposition peaked on day 6, coinciding with tubular injury and impaired kidney function in both wild‑type and IL‑37tg mice, as evidenced by reduced *t*GFR and elevated BUN, creatinine, and SDMA. Stone formation triggered a robust inflammatory response, with 46 of 84 genes upregulated > 3‑fold in wild‑type mice. IL‑37 expression did not alter crystal burden, kidney injury, kidney function, or inflammatory gene expression. These findings establish a refined murine CaOx model incorporating kidney function and molecular endpoints and demonstrate that IL‑37 does not confer renal protection in glyoxylate‑induced stone disease.

**Supplementary Information:**

The online version contains supplementary material available at https://doi.org/10.1007/s00240-026-02061-7.

## Introduction

Kidney stones are a common and recurrent urinary tract disorder, with a rising global incidence and a lifetime risk of up to 270 per 100,000 individuals [1, 2]. Among those with a history of kidney stones, nearly 50% experience recurrence within five years of the initial episode [1]. Calcium oxalate (CaOx), often mixed with calcium phosphate, constitutes approximately 80% of all stone cases [3].

In mice, intraperitoneal administration of glyoxylate induces renal CaOx crystal deposition, making it the most widely used model of CaOx nephropathy [4, 5]. CaOx stone formation occurs within renal tubules through CaOx crystal aggregation and growth [1]. Despite its widespread application, this model has not been extensively investigated with respect to the cytokine-mediated inflammatory response during CaOx crystal formation. Additionally, many studies lack standardized and quantitative assessments of crystal burden and kidney function, with crystal analysis often limited to representative images, rather than whole kidney measurements [4–6].

Kidney function is typically evaluated by ex vivo blood urea nitrogen (BUN) and serum creatinine, which rise after crystal formation but show high variability and poor reproducibility [6]. Symmetric dimethylarginine (SDMA) is an early blood marker of kidney function decline in rodents [7], and real-time, non-invasive assessment of in vivo kidney function has not yet been established for kidney stone models. To address this, we adopted a transdermal glomerular filtration rate (*t*GFR) measurement technique that enables accurate, real-time assessment of kidney function in conscious, freely moving mice. This technique has been successfully used in other models of renal injury and offers a physiologically relevant alternative to conventional ex vivo methods [8, 9].

Interleukin-37 (IL-37), a member of the interleukin-1 (IL-1) cytokine family, is a natural suppressor of inflammatory responses [10]. The IL-37 gene comprises six exons encoding five isoforms (IL-37a, b, c, d, and e) [11, 12]. IL-37 expression is induced following inflammatory stimuli [13], and in the human kidney, tubular epithelial cells express IL-37, which downregulates key pro-inflammatory mediators, including TNF, IL-6, and IL-1β [14]. Transgenic expression or recombinant administration of exogenous IL-37 confers protection against diverse inflammatory conditions in mice, including endotoxin shock, colitis, lung injury, spinal cord injury, coronary disease, arthritis, and inflammation-related fatigue, partly through its interaction with the IL-1 family receptor IL-1R8 [13, 15]. Notably, IL-37b, the isoform predominantly expressed in the kidney, attenuates disease progression in autosomal dominant polycystic kidney disease by enhancing interferon signaling pathways originating from kidney resident macrophages [16].

Given the potent anti-inflammatory activity of IL-37 and its renal expression, we hypothesized that IL-37 may protect against CaOx nephropathy and associated inflammation. To further enhance experimental rigor and reproducibility, we applied quantitative stone burden analysis, real-time longitudinal kidney function monitoring using *t*GFR, and broad profiling of inflammatory gene expression in the kidney. These complementary approaches aimed to define the role of IL-37 in CaOx crystal-induced renal injury and advance understanding of the immunopathogenesis of kidney stone disease.

## Methods

### Mice

6-8-week-old male C57BL/6Jax wild-type and IL-37tg mice were used [10]. All animals were placed under standard laboratory conditions with *ad libitum* access to water and a standard chow diet. Before experimentation, all animals were acclimatized for 1 week and housed in individually ventilated cages under specific pathogen-free, physical containment 2 conditions.

### Induction of calcium oxalate crystals

The CaOx mouse model was induced by daily intraperitoneal injection (i.p.) of 100 mg/kg (pH 7.4) glyoxylic acid monohydrate, also known as glyoxylate (Sigma-Aldrich, St. Louis, United States). 200 µL of 0.9% normal saline was used as a vehicle control. Glyoxylate stock concentration was prepared freshly for each experiment and kept at 4°C until administration. The working concentration of glyoxylate was prepared each day before i.p. injection.

### Quantification of CaOx crystals and tubular injury

Left kidneys were fixed in 4% formaldehyde solution (Sigma-Aldrich) before paraffin embedding and sectioning (4 μm). For histological assessment of stone formation, longitudinal kidney sections were stained with Pizzolato [4, 17] and quantified using Image J 1.49v software. A threshold ranging from 140 to 170 was set to isolate the CaOx crystals from background tissue. Tubular injury scoring was performed by a blinded investigator using an established semi-quantitative method [18] with some modifications, as previously described, using Periodic acid-Schiff (PAS) staining [18–20]. A score of 0 = 0% of tubules were affected; 1 = 1–10%; 2 = 11–25%; 3 = 26–50%; 4 = 51–75%; 5 = 76–100%. Representative sections were imaged using a Zeiss Axio microscope with ZEN 2 Software (Carl Zeiss Microscopy, New York, United States).

### Kidney function

 In vivo* t*GFR was assessed before and after induction of CaOx crystals using the MediBeacon system (MediBeacon gmbH, Mannheim, Germany) as described [9, 21]. In brief, two days before measurements, fur was removed from the dorsal side of the mouse using an electric razor and depilation cream, and a mini transdermal GFR monitor was attached above the right flank muscle. Steady-state fluorescence was measured for 3 min. An intravenous bolus of 50 mg/kg FITC-sinistrin (MediBeacon) was administered *via* the tail vein, and fluorescence decay was measured for 90 min. Data were analyzed using MB Studio 2 software using a three-compartment model with linear baseline correction.

### Serum BUN, creatinine and SDMA

Blood was collected by cardiac puncture, left to clot at room temperature for 30 min and centrifuged at 3,000 x *g* for 10 min at 4°C. Serum supernatants were stored at -80°C. BUN, serum creatinine, and SDMA were measured using individual slides on a Catalyst One veterinary chemistry analyzer (IDEXX Laboratories, Rydalmere, Australia).

### RNA extraction and reverse transcription

Total RNA was extracted from the right kidneys using mechanical homogenization, followed by TRIzol-based extraction as previously described [21, 22]. RNA quantity and purity were assessed by spectrophotometry, and samples were DNase-treated before reverse transcription using a standard MMLV protocol to generate cDNA for downstream qPCR analysis.

### RT2 Profiler PCR Arrays

RT^2^ profiler mouse inflammatory response and autoimmunity arrays were performed using Qiagen kit (QIAGEN, cat# 330231 PAMM-077Z), and data were analyzed using the Qiagen GeneGlobe platform for RT2 assays, as previously described [21].

### Gene expression analysis

Gene expression was quantified using SYBR Green PowerUp Mix assays (Thermo-Fisher Scientific). The relative gene expression was determined using the 2^−ΔΔCT^ method, with normalization to the geometric mean of the housekeeping gene, glyceraldehyde-3-phosphate dehydrogenase (*Gapdh*) [21–23].

### Statistical analysis

Analyses were performed using GraphPad Prism software version 10.5.0. An unpaired non-parametric Mann-Whitney test was used to compare two groups. Pearson correlation and simple linear regression were used for correlations. Details are provided in each figure legend.

### Ethics statement

Experiments were performed in accordance with the National Health and Medical Research Council Australian Code of Practice approved by the Alfred Research Alliance Animal Ethics Committee (P8003) and the Animal Care and Ethics Committee, University of Newcastle (A-2018-809).

## Results

### Glyoxylate-induced calcium oxalate crystal deposition peaks at day 6 and correlates with kidney injury in wild-type mice

**Fig. 1:**
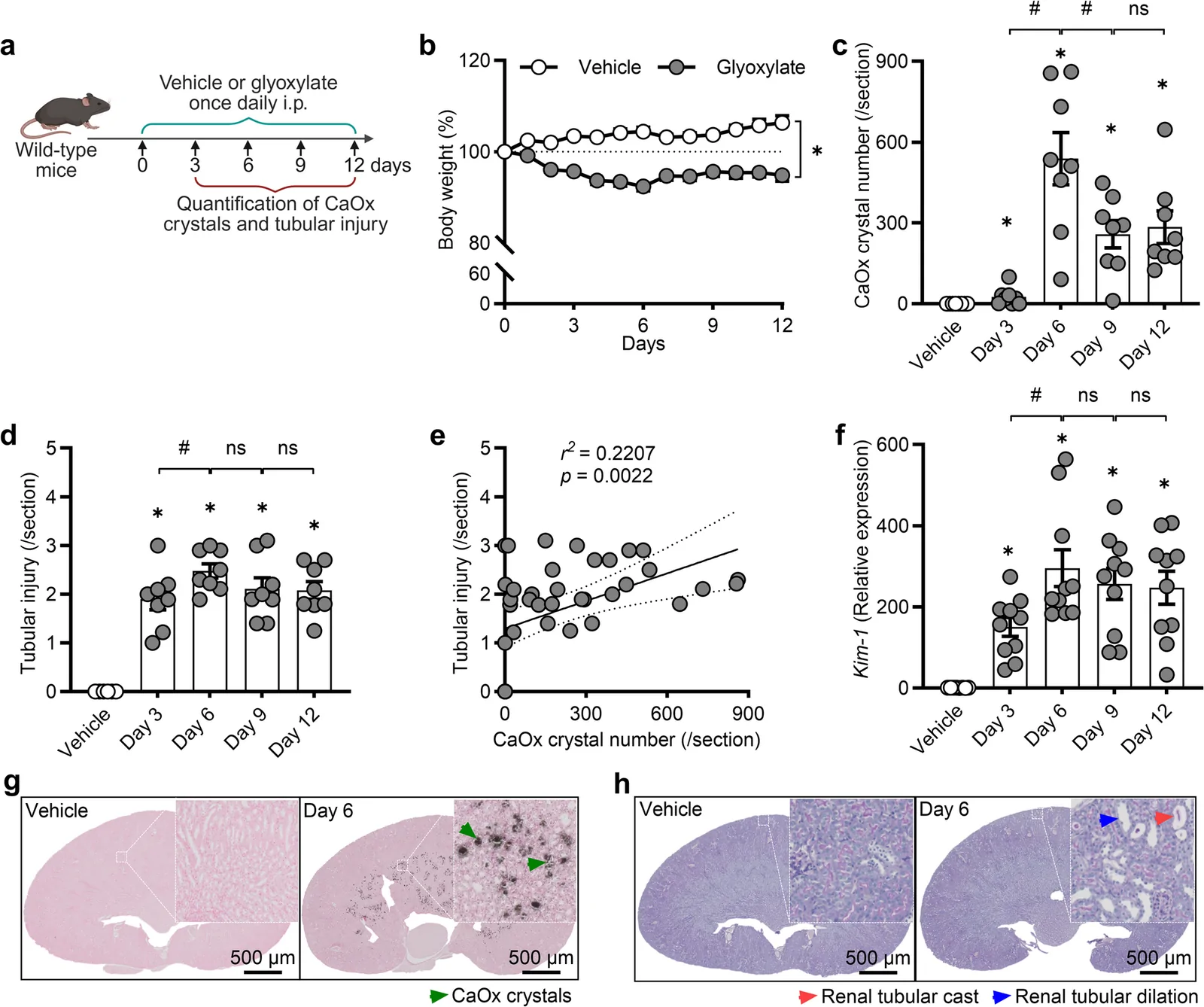
Glyoxylate-induced calcium oxalate crystal deposition peaks at day 6 and correlates with kidney injury in wild-type mice. (**a**) Schematic representation of the glyoxylate-induced calcium oxalate (CaOx) kidney stone model. (**b**) Body weight change over time. (**c**) Quantification of CaOx crystal numbers per kidney section. (**d**) Semi-quantitative scoring of tubular injury per kidney section. (**e**) Correlation between CaOx crystal number and tubular injury at day 6. (**f**) Kidney injury molecule 1 (*Kim-1*) mRNA expression in whole kidney homogenates. (**g**) Representative pizzolato-stained kidney sections showing CaOx crystal deposition at day 6. (**h**) Representative periodic acid-Schiff (PAS)-stained kidney sections showing tubular injury at day 6. Scale bar represents 500 μm. Data were obtained from two independent experiments. Data are presented as mean ± standard error of the mean (SEM). Sample size was *n* = 8–10 per time point and per group. Statistical analysis was performed using the non-parametric unpaired Mann-Whitney test. *P* value < 0.05. * denotes a statistical difference compared to vehicle controls. ^#^ denotes statistical differences between two consecutive time points. ns denotes not significant. Correlation analysis was performed using Pearson correlation and linear regression

CaOx crystals were induced by daily intraperitoneal administration of glyoxylate (Fig. 1a). Glyoxylate administration resulted in mild body weight loss over time (Fig. 1b). Significant CaOx crystal deposition was observed at days 3, 6, 9, and 12 in glyoxylate-treated mice, compared to vehicle-treated controls, with peak crystal accumulation occurring at day 6 (Fig. 1c). Glyoxylate treatment also increased tubular injury throughout the time course, which was most severe at day 6 (Fig. 1d). A modest positive correlation was observed between the number of CaOx crystals and tubular injury (Fig. 1e). Kidney injury molecule-1 (*Kim-1*) mRNA expression was increased at days 3, 6, 9, and 12 in glyoxylate-treated mice, compared to vehicle-treated controls, with maximum *Kim-1* expression occurring at day 6 (Fig. 1f). Furthermore, BUN and SDMA levels increased at days 3, 6, 9, and 12 in glyoxylate-treated mice, compared to vehicle-treated controls, with peak BUN and SDMA levels occurring at day 6 (**Supplementary Fig. 1a-c**). Crystals were primarily deposited in the cortico-medullary junction and medullary regions (Fig. 1g). Tubular injury, characterized by dilation and cast formation, was found in the glyoxylate-treated mice compared to vehicle controls (Fig. 1h). An analysis of 84 mouse inflammatory genes showed that 46 genes were upregulated by more than 3-fold at day 6 in glyoxylate-treated mice compared with vehicle-treated controls (Table 1). Collectively, day 6 was confirmed as the peak of CaOx crystal deposition, renal injury and inflammatory gene expression and was therefore selected for further investigation.

**Table 1:** List of up and down regulated genes at weeks 3, 6, and 9 in glyoxylate-induced CaOx stones in wild-type mice compared to saline-treated vehicle controls (Fold change, pooled samples; *n* = 9–10 per group)

Gene Symbol	Description	Fold change (Relative to wild-type saline)		
		Day 3 GOx	Day 6 GOx	Day 9 GOx
*Bcl6*	B-cell leukemia/lymphoma 6	1.06	1.59	1.16
*C3*	Complement component 3	21.87	35.78	21.91
*C3ar1*	Complement component 3a receptor 1	2.47	5.76	5.43
*C4b*	Complement component 4B (Chido blood group)	8.69	10.87	9.82
*Ccl1*	Chemokine (C-C motif) ligand 1	1.33	7.17	3.04
*Ccl11*	Chemokine (C-C motif) ligand 11	7.44	10.87	4.24
*Ccl12*	Chemokine (C-C motif) ligand 12	10.53	38.94	30.73
*Ccl17*	Chemokine (C-C motif) ligand 17	2.04	5.46	5.47
*Ccl19*	Chemokine (C-C motif) ligand 19	1.87	2.72	1.98
*Ccl2*	Chemokine (C-C motif) ligand 2	8.41	24.50	25.32
*Ccl20*	Chemokine (C-C motif) ligand 20	30.09	13.64	19.01
*Ccl22*	Chemokine (C-C motif) ligand 22	1.42	3.30	2.60
*Ccl24*	Chemokine (C-C motif) ligand 24	1.56	1.31	0.90
*Ccl25*	Chemokine (C-C motif) ligand 25	2.06	0.60	2.40
*Ccl3*	Chemokine (C-C motif) ligand 3	1.94	3.19	3.72
*Ccl4*	Chemokine (C-C motif) ligand 4	1.62	2.59	2.27
*Ccl5*	Chemokine (C-C motif) ligand 5	2.40	2.95	3.65
*Ccl7*	Chemokine (C-C motif) ligand 7	4.91	20.05	30.32
*Ccl8*	Chemokine (C-C motif) ligand 8	5.11	17.06	30.69
*Ccr1*	Chemokine (C-C motif) receptor 1	5.09	10.50	6.71
*Ccr2*	Chemokine (C-C motif) receptor 2	3.06	6.47	7.38
*Ccr3*	Chemokine (C-C motif) receptor 3	4.51	12.47	9.80
*Ccr4*	Chemokine (C-C motif) receptor 4	0.92	0.23	0.70
*Ccr7*	Chemokine (C-C motif) receptor 7	1.17	3.49	6.44
*Cd14*	CD14 antigen	11.31	10.22	15.71
*Cd40*	CD40 antigen	2.55	4.06	3.36
*Cd40lg*	CD40 ligand	1.69	1.31	0.84
*Cebpb*	CCAAT/enhancer binding protein (C/EBP), beta	2.57	3.66	2.59
*Crp*	C-reactive protein, pentraxin-related	1.33	1.31	0.87
*Csf1*	Colony stimulating factor 1 (macrophage)	3.03	5.83	4.03
*Cxcl1*	Chemokine (C-X-C motif) ligand 1	23.45	82.43	51.81
*Cxcl10*	Chemokine (C-X-C motif) ligand 10	7.13	9.27	6.68
*Cxcl11*	Chemokine (C-X-C motif) ligand 11	0.16	0.16	0.13
*Cxcl2*	Chemokine (C-X-C motif) ligand 2	82.61	227.45	39.25
*Cxcl3*	Chemokine (C-X-C motif) ligand 3	1.61	5.00	1.07
*Cxcl5*	Chemokine (C-X-C motif) ligand 5	6.49	6.33	15.82
*Cxcl9*	Chemokine (C-X-C motif) ligand 9	0.13	0.69	0.65
*Cxcr1*	Chemokine (C-X-C motif) receptor 1	1.33	1.31	0.84
*Cxcr2*	Chemokine (C-X-C motif) receptor 2	1.45	1.31	4.31
*Cxcr4*	Chemokine (C-X-C motif) receptor 4	2.02	3.68	3.38
*Fasl*	Fas ligand (TNF superfamily, member 6)	1.33	1.31	0.84
*Fos*	FBJ osteosarcoma oncogene	4.15	6.76	6.03
*Ifng*	Interferon gamma	2.06	0.79	0.13
*Il10*	Interleukin 10	1.28	11.17	1.06
*Il10rb*	Interleukin 10 receptor, beta	1.37	2.17	1.67
*Il17a*	Interleukin 17 A	1.33	1.31	0.84
*Il18*	Interleukin 18	1.91	3.41	2.23
*Il1a*	Interleukin 1 alpha	9.05	12.76	17.28
*Il1b*	Interleukin 1 beta	1.34	4.02	4.00
*Il1r1*	Interleukin 1 receptor, type I	1.97	3.07	2.89
*Il1rap*	Interleukin 1 receptor accessory protein	1.79	0.99	1.19
*Il1rn*	Interleukin 1 receptor antagonist	23.74	36.68	33.48
*Il22*	Interleukin 22	1.33	1.31	0.84
*Il23a*	Interleukin 23, alpha subunit p19	0.74	0.56	0.39
*Il23r*	Interleukin 23 receptor	15.70	72.41	24.48
*Il5*	Interleukin 5	1.65	0.32	1.06
*Il6*	Interleukin 6	24.00	26.45	14.93
*Il6ra*	Interleukin 6 receptor, alpha	1.77	3.03	3.26
*Il7*	Interleukin 7	1.49	1.30	1.21
*Il9*	Interleukin 9	1.33	1.72	3.82
*Itgb2*	Integrin beta 2	4.43	5.71	7.14
*Kng1*	Kininogen 1	2.63	3.49	7.31
*Lta*	Lymphotoxin A	0.40	0.17	0.08
*Ltb*	Lymphotoxin B	1.75	1.75	1.32
*Ly96*	Lymphocyte antigen 96	1.35	1.69	1.60
*Myd88*	Myeloid differentiation primary response gene 88	2.30	2.12	2.20
*Nfkb1*	Nuclear factor of kappa light polypeptide gene enhancer in B-cells 1, p105	1.39	2.69	2.32
*Nos2*	Nitric oxide synthase 2, inducible	0.98	1.13	0.77
*Nr3c1*	Nuclear receptor subfamily 3, group C, member 1	1.29	1.28	1.07
*Ptgs2*	Prostaglandin-endoperoxide synthase 2	2.56	2.41	1.74
*Ripk2*	Receptor (TNFRSF)-interacting serine-threonine kinase 2	1.75	1.70	1.53
*Sele*	Selectin, endothelial cell	3.47	4.31	1.74
*Tirap*	Toll-interleukin 1 receptor (TIR) domain-containing adaptor protein	2.37	2.56	2.22
*Tlr1*	Toll-like receptor 1	8.26	12.19	15.15
*Tlr2*	Toll-like receptor 2	3.71	4.93	3.93
*Tlr3*	Toll-like receptor 3	0.45	1.11	1.06
*Tlr4*	Toll-like receptor 4	1.94	2.10	3.19
*Tlr5*	Toll-like receptor 5	3.91	6.87	5.52
*Tlr6*	Toll-like receptor 6	0.28	1.47	3.22
*Tlr7*	Toll-like receptor 7	0.77	8.48	10.59
*Tlr9*	Toll-like receptor 9	1.37	3.42	3.12
*Tnf*	Tumor necrosis factor	5.38	12.95	5.18
*Tnfsf14*	Tumor necrosis factor (ligand) superfamily, member 14	1.43	1.31	0.84
*Tollip*	Toll interacting protein	1.40	1.41	1.09

### Glyoxylate-induced calcium oxalate nephropathy leads to a significant decline in kidney function in wild-type mice

**Fig. 2:**
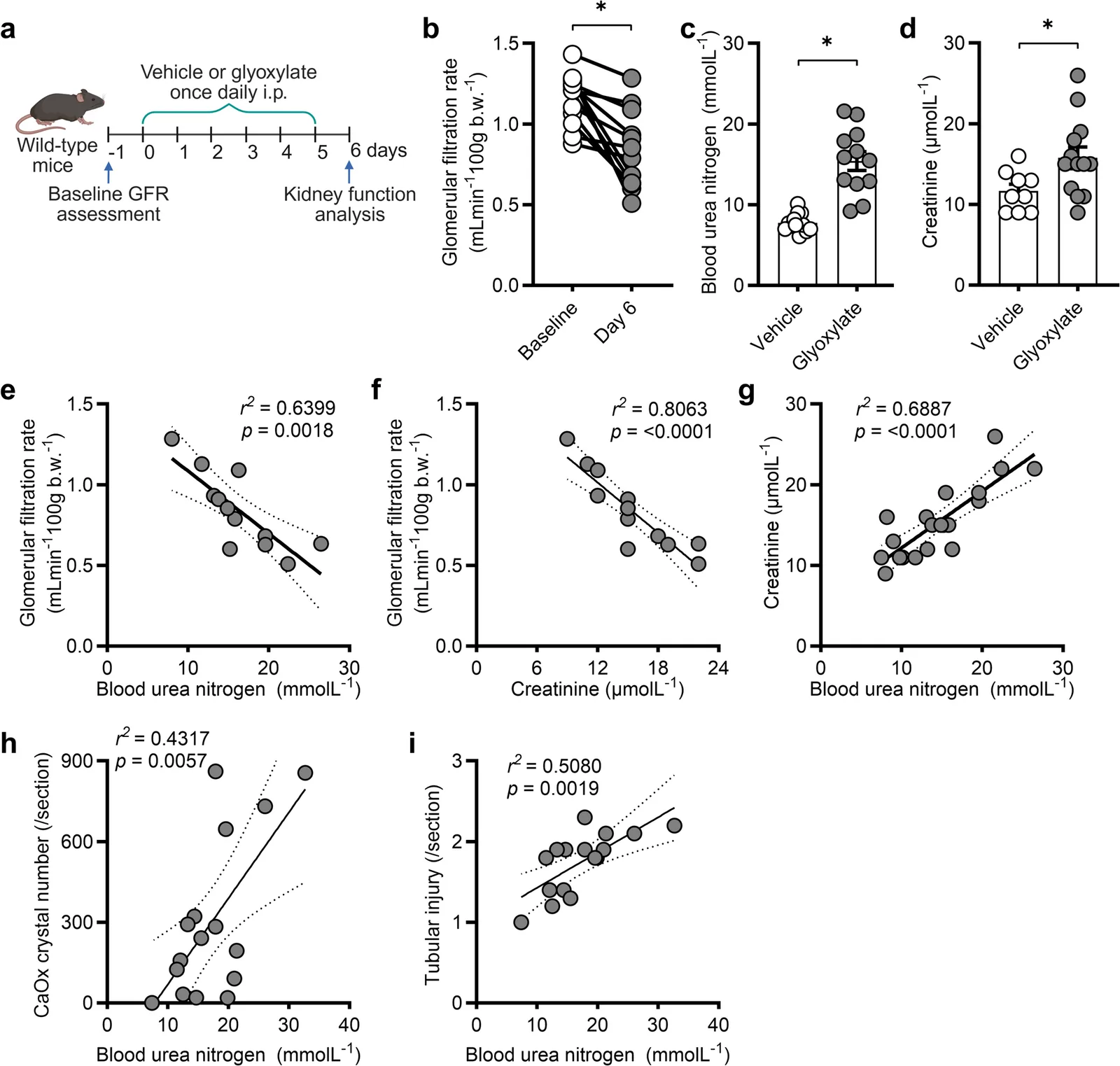
Glyoxylate-induced calcium oxalate nephropathy leads to a significant decline in kidney function in wild-type mice. (**a**) Schematic representation of transdermal glomerular filtration rate (*t*GFR) measurement in the calcium oxalate (CaOx) kidney stone model. (**b**) Paired *t*GFR measurements before (baseline) and after glyoxylate administration (Day 6) in wild-type mice (*n* = 12). (**c**) Serum blood urea nitrogen (BUN) levels at day 6 (*n* = 12 per group). (**d**) Serum creatinine levels at day 6 (*n* = 9–13 per group). (**e-i**) Correlations between *t*GFR and BUN (*n* = 12), *t*GFR and creatinine (*n* = 12), BUN and creatinine (*n* = 20), CaOx crystals and BUN (*n* = 16), and tubular injury and BUN (*n* = 16) at day 6. Data were obtained from two independent experiments and are presented as mean ± the standard error of the mean (SEM). Statistical analysis was performed using the Mann-Whitney t-test. *P* value < 0.05. * denotes a statistical difference compared to vehicle controls. Correlations were assessed using Pearson correlation and linear regression

Real-time in vivo* t*GFR was measured at baseline, before glyoxylate administration, and again on day 6 at the peak of CaOx crystal formation (Fig. 2a). Glyoxylate administration resulted in a significant reduction in *t*GFR at day 6 compared to baseline in the same animals (Fig. 2b). Additionally, BUN and serum creatinine levels were significantly elevated in glyoxylate-treated mice at day 6 relative to vehicle-treated controls (Fig. 2c-d). A strong negative correlation was observed between *t*GFR and BUN (Fig. 2e) and between *t*GFR and serum creatinine (Fig. 2f), indicating impaired renal function. Conversely, BUN and serum creatinine levels were positively correlated at day 6 in glyoxylate-treated mice (Fig. 2g). Both CaOx crystal number and tubular injury positively correlated with BUN (Fig. 2h-i).

### IL-37 does not significantly affect kidney injury in a mouse model of CaOx crystal formation at day 6

**Fig. 3:**
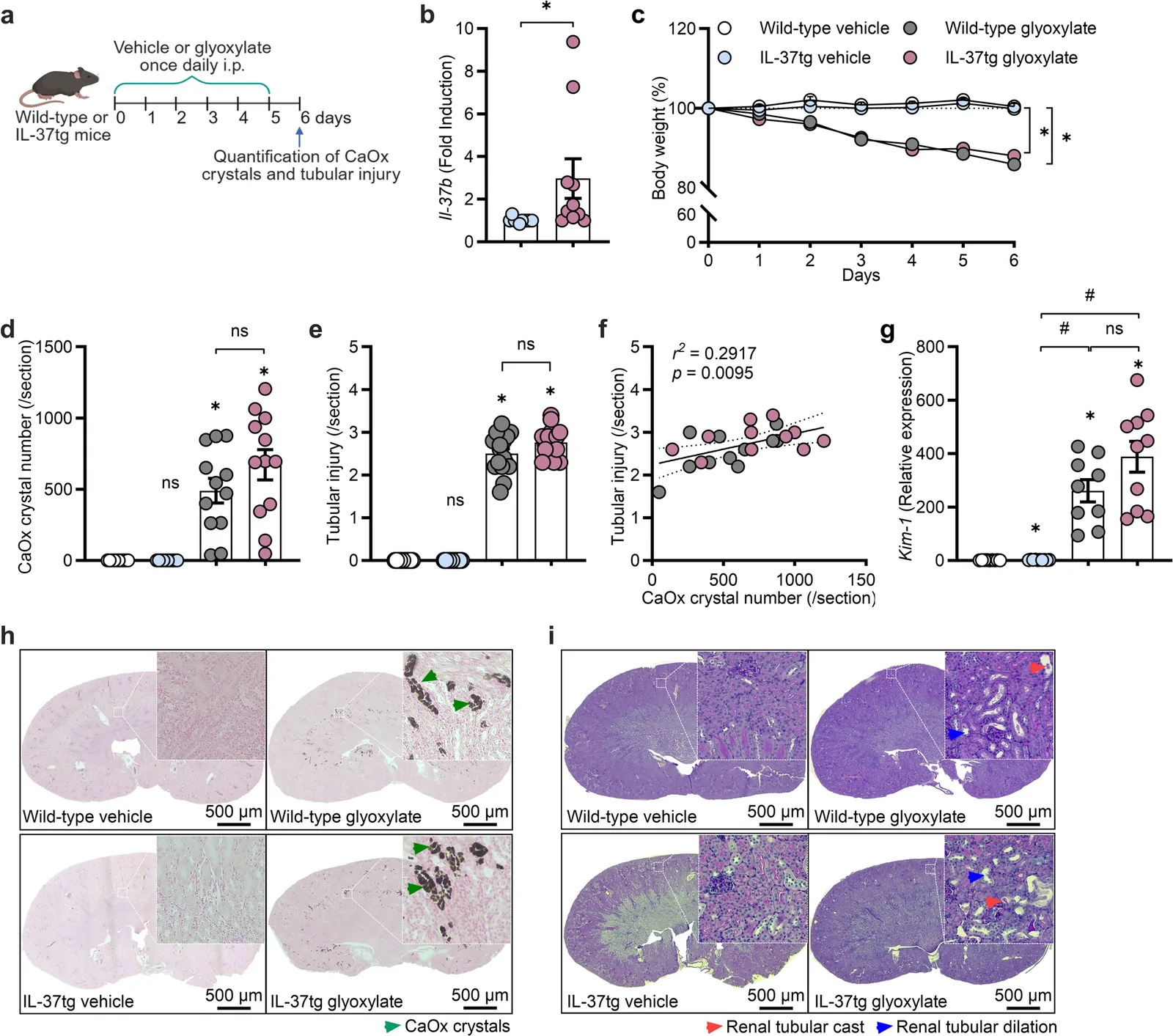
IL-37 does not significantly affect kidney injury in a mouse model of CaOx crystal formation at day 6. (**a**) Schematic representation of glyoxylate-induced calcium oxalate (CaOx) kidney stone model. (**b**) Kidney *Il37b* mRNA expression in glyoxylate- and vehicle-treated IL-37tg mice (*n* = 10–12 per group). (**c**) Body weight change over time. (**d**) Quantification of CaOx crystal numbers per kidney section (*n* = 10–12 per group). (**e**) Tubular injury scores per kidney section (*n* = 12–16 per group). (**f**) Correlation between crystal number and tubular injury (*n* = 11 per group). (**g**) *Kim-1* mRNA expression (*n* = 9–10 per group). Representative (**h**) Pizzolato- and (**i**) periodic acid-Schiff (PAS)-stained kidney sections at day 6. Scale bar indicates 500 μm. Data were obtained from four independent experiments and are presented as mean ± the standard error of the mean (SEM). Gene expression values are normalized to the mean of the vehicle controls and presented as mean ± standard error of the mean (SEM). Statistical analysis was performed using the Mann-Whitney t-test; *P* value < 0.05. * denotes a statistical difference compared to vehicle controls. ^#^ denotes statistical differences between two groups. ns denotes not significant. Correlations were assessed using Pearson correlation and linear regression

Both wild-type and IL-37tg mice were administered glyoxylate daily (Fig. 3a). *IL37b* mRNA expression was significantly increased at day 6 in IL-37tg mice following glyoxylate administration compared to vehicle-treated IL-37tg controls (Fig. 3b). Glyoxylate treatment caused mild weight loss in both wild-type and IL-37tg mice compared to their respective vehicle-treated controls (Fig. 3c). CaOx crystal deposition in the kidney of IL-37tg mice was comparable to glyoxylate-treated wild-type controls (Fig. 3d). Additionally, tubular injury was similar in IL-37tg and wild-type controls (Fig. 3e). A modest positive correlation between tubular injury and crystal number was observed in both groups (Fig. 3f). *Kim-1* mRNA expression was similar between IL-37tg and wild-type controls (Fig. 3g). Crystal deposition consistently occurs in the cortico-medullary junction and medulla, regardless of mouse genotype (Fig. 3h). Tubular injury was also consistently localized to the cortex across groups, characterized by tubular dilation and cast formation (Fig. 3i).

### IL-37 does not significantly affect kidney inflammatory gene expression in a mouse model of CaOx crystal formation at day 6

**Fig. 4:**
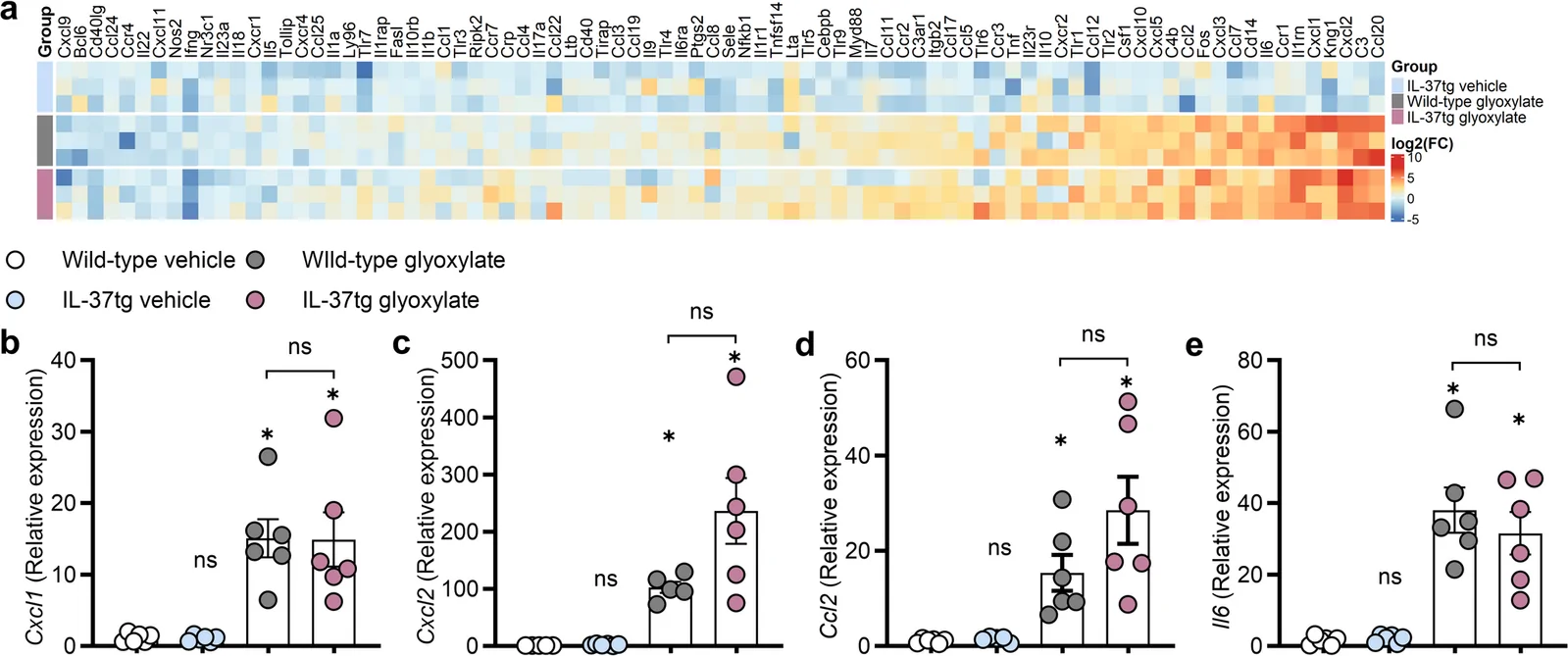
IL-37 does not significantly affect kidney inflammatory gene expression in a mouse model of CaOx crystal formation at day 6. Inflammatory gene expression was assessed in kidney homogenates of glyoxylate- and vehicle-treated wild-type and IL-37 transgenic (IL-37tg) mice. (**a**) Heat map of qPCR profiling of inflammatory gene transcripts in glyoxylate- and vehicle-treated wild-type and IL-37tg kidney homogenates, depicted as log2-transformed fold change compared to vehicle-treated wild-type controls (*n* = 3 per group). (**b-e**) Targeted qPCR analysis of chemokine (C-X-C motif) ligand 1 (*Cxcl1*), *Cxcl2*, chemokine (C-C motif) ligand 2 (*Ccl2*), and interleukin 6 (*Il6*) (*n* = 6 per group). *Gapdh* was used as the housekeeping gene. Data are shown as mean ± standard error of the mean (SEM), normalized to the mean of the wild-type vehicle-treated control group. Statistical significance was determined using a non-parametric unpaired Mann–Whitney test. *P* value < 0.05. * denotes a significant difference compared to vehicle controls. ns denotes not significant

Administration of glyoxylate in IL-37tg mice induced comparable upregulation of inflammatory genes in the kidney compared to glyoxylate-treated wild-type controls. (Fig. 4a). The relative expression of *Cxcl1*, *Cxcl2*, *Ccl2*, and *Il6* increased in both wild-type and IL-37tg glyoxylate-treated mice compared to their respective vehicle-treated controls (Fig. 4b-e). There were no significant differences in kidney inflammatory gene expression in IL-37tg glyoxylate-treated mice compared to wild-type glyoxylate-treated controls (Fig. 4b-e).

### IL-37 does not significantly affect kidney function in a mouse model of CaOx crystal formation at day 6

**Fig. 5:**
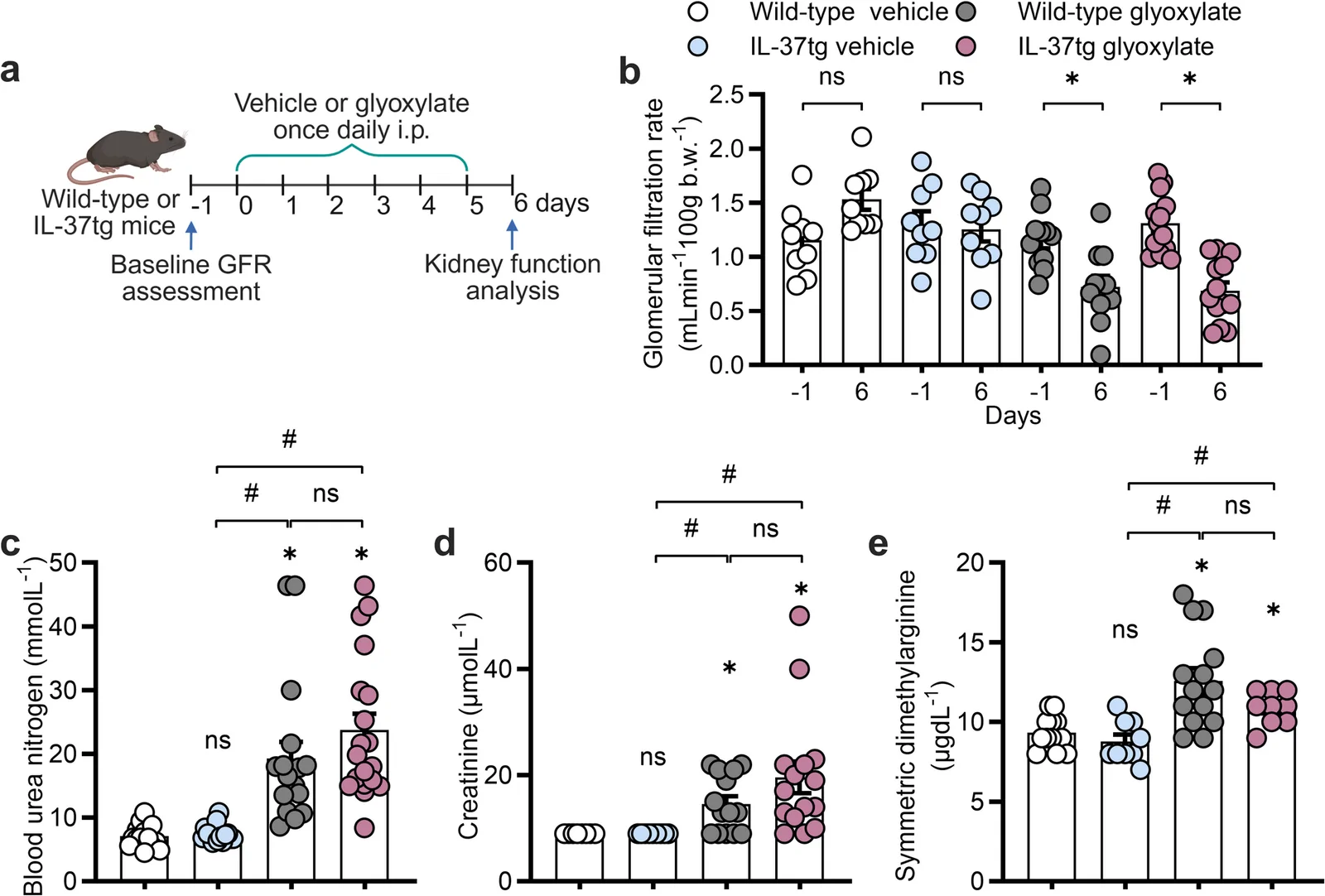
IL-37 does not significantly affect kidney function in a mouse model of CaOx crystal formation at day 6. (**a**) Schematic representation of transdermal glomerular filtration rate (*t*GFR) measurement in the calcium oxalate (CaOx) kidney stone model. (**b**) Paired *t*GFR measurements before (baseline) and after (day 6) vehicle or glyoxylate administration (*n* = 8–13 per group). (**c**) Blood urea nitrogen (BUN) levels at day 6 (*n* = 15–19 per group). (**d**) Serum creatinine levels at day 6 (*n* = 8–15 per group). (**e**) Serum symmetric dimethylarginine (SDMA) levels at day 6 (*n* = 10–14 per group). Data were obtained from four independent experiments and are shown as mean ± the standard error of the mean (SEM). Statistical analysis was performed using the Mann-Whitney t-test; *P* value < 0.05. * denotes a statistical difference compared to vehicle controls. ^#^ denotes statistical differences between two groups as shown. ns denotes not significant

Paired *t*GFR measurements were performed at baseline and day 6 following glyoxylate treatment in wild-type and IL-37tg mice (Fig. 5a). Both wild-type and IL-37tg glyoxylate-treated mice exhibited a significant decline in *t*GFR at day 6 compared to their respective baselines (Fig. 5b). However, there was no significant difference in *t*GFR between the two genotypes (Fig. 5b). BUN, serum creatinine, and SDMA levels were significantly increased in glyoxylate-treated wild-type and IL-37tg mice at day 6 compared to their respective vehicle-treated controls (Fig. 5c-e). No significant difference in BUN, creatinine or SDMA was observed in glyoxylate-treated IL-37tg mice compared to glyoxylate-treated wild-type controls (Fig. 5c-e).

## Discussion

Consistent with previous reports, we successfully replicated the glyoxylate-induced CaOx mouse model of kidney stone disease using daily intraperitoneal glyoxylate administration [4]. In this study, we defined the peak time point of CaOx crystal formation, quantified crystal deposition, and assessed kidney function and renal inflammation using in vivo and molecular readouts. We also evaluated the therapeutic potential of the anti-inflammatory cytokine IL-37 in this context.

When CaOx crystals are formed in the kidney, smaller crystals are typically excreted in the urine, whereas larger crystals remain embedded within renal tubules, contributing to kidney injury. Unlike previous studies that primarily reported the presence or absence of crystals or analyzed crystal area in randomly selected kidney [4–6, 24], we report quantification of CaOx crystals in whole kidney sections. We observed that CaOx crystal burden peaked at day 6 post-glyoxylate injection, aligning with previous reports [4, 25]. This peak crystal deposition correlated with notable tubular changes, including dilation, cast formation, and injury in the cortex and cortico-medullary junction [5].

To assess impaired kidney function in this model, we used both conventional ex vivo markers, BUN and serum creatinine, and an in vivo real-time *t*GFR. While BUN and creatinine are standard biomarkers, they have limitations in sensitivity and specificity [6, 26]. To address these limitations, we adopted the *t*GFR method, which has been validated as a reliable tool in mouse models of acute kidney injury [9]. This minimally invasive approach enabled real-time, longitudinal assessment of kidney function without the need for blood or urine collection or surgical catheterization. Our data showed a decline in *t*GFR at day 6, coinciding with peak stone formation, suggesting impaired kidney function. These measurements were reproducible and strongly correlated with changes in BUN and serum creatinine, highlighting the robustness of *t*GFR for measuring kidney function in vivo [27, 28]. However, it is essential to note that glyoxylate administration may also have nephrotoxic effects, potentially impairing kidney function. To address this, we show that both the number of CaOx crystals and tubular injury correlate positively with BUN, suggesting that increasing stone burden and renal injury in this model are associated with impaired kidney function rather than with glyoxylate renal toxicity alone. Further evidence for CaOx crystal-induced injury comes from the upregulation of *Kim-1* mRNA, a well-established marker of kidney injury [29]. Additional markers, such as Neutrophil gelatinase-associated lipocalin (NGAL) and albuminuria, may also detect subclinical differences that global measures like *t*GFR, BUN, or creatinine can miss, underscoring the value of incorporating these readouts in future studies. To address this, we assessed serum SDMA levels as an early marker of kidney function decline. Serum SDMA showed similar changes to those of *t*GFR, BUN, and creatinine. Urinary chemistry parameters, such as oxalate, citrate, pH, calcium, and osmolality, were not assessed in this study, and their inclusion in future studies would enhance translational relevance by enabling closer alignment with human lithogenic conditions.

To characterize the inflammatory response associated with CaOx stone formation, we profiled the expression of cytokine and chemokine genes in the kidneys of mice. Of the 84 genes assessed, 46 were altered by more than 3-fold at day 6, highlighting the strong inflammatory phenotype of this model. This aligns with previous studies showing increased inflammation in both mouse models and human CaOx stone disease [5, 30, 31]. Consistent with these reports, we observed marked upregulation of pro-inflammatory mediators, including *Cxcl1*, *Cxcl2*, *Ccl2*, and *Il6*, in the kidneys [32, 33]. This inflammatory response set the stage for exploring the role of the anti-inflammatory cytokine IL-37 in CaOx nephropathy. IL-37 is known to be protective in various inflammatory and metabolic disorders, such as colitis, cancer, and arthritis [15, 34, 35]. However, transgenic expression of human IL-37 in mice did not significantly alter CaOx crystal formation, kidney function, or inflammatory gene expression. Together, these findings indicate that IL-37 does not affect the CaOx crystal burden, tubular injury, or kidney function at the peak of disease in this model. Additionally, glyoxylate-induced renal proinflammatory and kidney injury marker gene expression may be resistant to IL-37-mediated suppression under these experimental conditions. Although IL-37 did not confer protection against stone formation, kidney injury, or impaired kidney function at the peak of CaOx injury (day 6), this represents only one stage of disease. IL-37 may act earlier (24–72 h) during crystal nucleation and early inflammation, or later (day 9–12) during resolution and repair. Our findings should therefore be interpreted as time-point-specific, and additional early- and late-phase assessments will be needed to determine whether IL-37 influences other phases of stone pathogenesis. Assessing IL-37 protein abundance, tissue localization, processing status, and receptor co-expression would provide additional information on transgene expression. However, reliable detection of IL-37 protein is technically challenging, especially in tissues, as IL-37 is expressed at low levels and can be difficult to detect using conventional methods [36]. We therefore used *Il37b* mRNA expression as a robust and reproducible measure of transgene expression in this study.

Indeed, most evidence supporting the therapeutic efficacy of IL‑37 comes from acute inflammation models, including necrotizing enterocolitis (≈ 3 days), endotoxemia (≈ 24 h), gout (≈ 4 h), and ischemic stroke (1–3 days) [10, 37–40]. In contrast, data from longer‑term or chronic disease models remain limited, likely because IL‑37 is regulated by several endogenous mechanisms that rapidly constrain its activity, including mRNA instability elements and post‑translational modifications such as homodimerization, which attenuate its bioactivity [41, 42]. IL‑37 also functions as an alarmin, characterized by rapid release and short‑lived action [43].

Existing evidence for IL‑37 in chronic inflammatory settings includes murine models of allergic asthma extending up to ~ 12 weeks and intestinal inflammation models lasting from several days to approximately two months [44, 45]. Importantly, this discrepancy does not necessarily indicate that IL‑37 lacks efficacy in chronic disease; rather, it reflects the practical and biological challenges inherent to studying IL‑37 in prolonged experimental systems. Furthermore, when IL-37 is processed by neutrophil elastase or cathepsin S, it can signal through IL-1R6 and gain pro-inflammatory functions [46].

In summary, this study advances the characterization of the glyoxylate-induced CaOx kidney stone model in mice by incorporating methods for quantifying CaOx crystals in whole kidney sections, evaluating real-time in vivo kidney function, and broadly profiling inflammatory gene responses. While IL-37 did not show protective or anti-inflammatory effects in this context, our approach lays the groundwork for future mechanistic studies to identify therapeutic targets for kidney stone disease that are renoprotective and maintain kidney function. This is crucial for developing strategies to mitigate CaOx-induced renal injury and improve outcomes in stone-forming individuals.

## Supplementary Information

Below is the link to the electronic supplementary material.

Supplementary Material 1

## Data Availability

All data supporting the findings of this study are included in this manuscript.
