# Supplementary material for: Interleukin-37 does not confer protection against stone formation or kidney injury at the peak of disease in a murine calcium oxalate model
Source: Urolithiasis. 2026 Oct 10;54(1):171. doi: 10.1007/s00240-026-02061-7 (PMC13653896; doi:10.1007/s00240-026-02061-7)
Supplement: Supplementary file 1 [file 240_2026_2061_MOESM1_ESM.docx]

**Interleukin-37 Does Not Confer Protection Against Stone Formation or Kidney Injury at the Peak of Disease in a Murine Calcium Oxalate Model**

Nirmal P. Bhatt^1, 2, 3^, Theresa T. H. Nguyen^1, 2^, Ina Rudloff^3, 4^, Bernadette Jones-Freeman^1^, Guy J. M. Cameron^5^, Gabriel R. Rodriguez^1, 2^, Marcel F. Nold^3, 4^, Aniruddh V. Deshpande^6, 7^, Claudia A. Nold-Petry^3, 4^, and Malcolm R. Starkey^1, 2, 8^*

^1^Department of Immunology, School of Translational Medicine, Monash University, Melbourne, Victoria, Australia

^2^Bladder and Kidney Health Discovery Program, School of Translational Medicine, Monash University, Melbourne, Victoria, Australia

^3^The Ritchie Centre, Hudson Institute of Medical Research, Melbourne, Victoria, Australia.

^4^Department of Paediatrics, Monash University, Melbourne, Victoria, Australia.

^5^Priority Research Centre GrowUpWell, College of Health, Medicine and Wellbeing, Hunter Medical Research Institute, The University of Newcastle, Newcastle, New South Wales, Australia

^6^Centre for Kidney Research, The Children’s Hospital at Westmead, The University of Sydney, Sydney, New South Wales, Australia

^7^Urology Unit, Department of Surgery, The Children’s Hospital at Westmead, Sydney, New South Wales, Australia

^8^School of Medical Sciences, Faculty of Health and Medicine, The University of Sydney, Sydney, New South Wales, Australia

*Correspondence: Dr Malcolm Ronald Starkey

E: malcolm.starkey@monash.edu

**Supplementary results**

**Glyoxylate-induced renal dysfunction peaks at day 6**

CaOx crystals were induced by daily intraperitoneal administration of glyoxylate (**Fig. S1a**). Glyoxylate-treated mice exhibited significantly increased blood urea nitrogen (BUN) levels at days 3, 6, 9, and 12 compared with vehicle-treated controls, with peak levels observed at day 6 (**Fig. S1b**). Similarly, serum symmetric dimethylarginine (SDMA) levels were significantly increased on days 3, 6, 9, and 12 in glyoxylate-treated mice compared with vehicle-treated controls, also peaking on day 6 (**Fig. S1c**).

**Figure S1. Serum blood urea nitrogen and symmetric dimethylarginine peak at day 6.** (**a**) Schematic representation of the glyoxylate-induced calcium oxalate (CaOx) kidney stone model. (**b**) Serum blood urea nitrogen (BUN) levels at days 3, 6, 9, and 12. (**c**) Serum symmetric dimethylarginine (SDMA) levels at days 3, 6, 9, and 12. Data were obtained from two independent experiments and are presented as mean ± standard error of the mean (SEM). Sample size was *n* = 9-12 per time point and per group. Statistical analysis was performed using the non-parametric unpaired Mann-Whitney test. *P* value < 0.05. * denotes a statistically significant difference compared with vehicle controls. ^#^ denotes a statistically significant difference between two consecutive time points. ns denotes not significant.
